# The demography and disease burden of the homeless shelter population of Tshwane during COVID-19

**DOI:** 10.4102/phcfm.v14i1.3692

**Published:** 2022-12-20

**Authors:** Paul S. Germishuys, Selma Smith, Jannie Hugo, Edith Madela-Mntla, Tanita Botha

**Affiliations:** 1 Department of Family Medicine, Faculty of Health Sciences, University of Pretoria, Pretoria, South Africa; 2 Department of Statistics, Faculty of Agriculture, University of Pretoria, Pretoria, South Africa

**Keywords:** family medicine, primary healthcare, homeless population, COVID-19, Tshwane District, lockdown

## Abstract

**Background:**

Homeless people are a vulnerable population susceptible to physical and mental health care problems. There are, however, limited studies and information regarding the health of the homeless population.

**Aim:**

To describe and understand the burden of disease among the homeless population in Tshwane District, Gauteng, South Africa.

**Setting:**

Data were collected from 15 different homeless shelters created during the South African 2020 coronavirus disease 2019 (COVID-19) lockdown in the Tshwane District, from April to July 2020.

**Methods:**

A cross-sectional survey was conducted among the homeless people in the shelters to provide information of self-reported conditions that the homeless populations at the shelters had during the lockdown period. The participants were also screened for medical conditions like, human immunodeficiency virus (HIV), hypertension (HPT) and diabetes mellites (DM).

**Results:**

Results showed a total of 2066 homeless population out of which 1391 took part in the survey. Most of the participants consisted of African males 93.83%, with substance use prevalence in 52.77%. The study showed that the population was very reluctant to share information and had less chronic conditions than originally thought.

**Conclusion:**

Efforts should be made to improve education and research around the homeless population, by government and non-government facilities by building relationships with homeless shelters in their areas.

**Contribution:**

This study provides awareness of the homeless population’s health and challenges, with the intention to attempt a better understanding of the population that may present themselves to primary healthcare (PHC) facilities and encourage future investigation into how to improve care.

## Introduction

The homeless population are vulnerable and susceptible to physical and mental healthcare problems.^1,2,3^ The physical health problems could be partly because of their exposure to the elements during the different seasons of the year.^1^ The homeless may also be at higher risk of developing anxiety and depression because of lack of infrastructure and stability provided by having a home and/or family support.^1^

Some general beliefs held by communities and resulting responses impact negatively on the healthcare for the homeless and can lead to lack of mutual respect and quality of care given to homeless people. One general belief is that homeless people abuse substances.^1,3,4^ This may be true as substance abuse may lead to being rejected from a job or home, but being on the street, may also be a reason for a non-substance user to turn to the use of alcohol or other substances.^1^ This might be their coping mechanism. Another general belief is that all homeless have a history of some form of criminal activity in the past that led them to become homeless.^2^ This belief impacts their access to healthcare, as homeless people may be treated with less respect or with minimal effort.^1^ The homeless population that has been exposed to discrimination for being homeless may also have a lack of respect for healthcare. This increases the risk of defaulting treatments for chronic illnesses or even preventing them from coming to the healthcare services to screen for the possibility of having a chronic illness.^1^

There is a paucity of information on the physical and mental health problems of the homeless in South Africa as most studies on the homelessness are done in developed countries like the United States of America (USA) and very few have been done in South Africa.^1,5,6^ The continued mobility of homeless people also contributes to the difficulty of studying their healthcare needs.^7,8^ The few studies done in South Africa indicate that most homeless people are single males. They are mobile, only staying short periods of time in different areas.^5^ According to 2014 statistics, 32% of the homeless in Tshwane are originally from Gauteng province, 52% are from other provinces, and 15% are from outside South Africa.^8^ One South African study conducted in 2019 evaluated 178 homeless living in Johannesburg. The results showed that 17% had human immunodeficiency virus (HIV), 7% had previous tuberculosis (TB), and 31% had chronic conditions.^2^ The 2011 formal figures in the City of Tshwane (COT) suggest there are approximately 6244 homeless people with no alternative places to stay in Tshwane.^8^

South Africa’s response to the coronavirus disease 2019 (COVID-19) pandemic was to initiate lockdown. The national lockdown implied that no one would be allowed to walk around in the streets and created the need for the start of homeless shelters at different locations in South Africa. This led to placement of a mixture of homeless people in shelters as some of them were street-homeless with no home and others were day strollers with occasional shelter living, depending on the situation at the time.^9,10,11^

Co-operation between COT and the Department of Family Medicine, University of Pretoria (DFMUP) in providing medical care at the homeless shelters during the (COVID-19) lockdown created a temporary but ideal opportunity to gain a better understanding of the demographics and disease profile of the homeless population in the Tshwane District.^3,9,10,11^ The project drew together governmental organisations and the private sector.^10,11^ The gained knowledge should inform policies and support programmes addressing the homeless situation in future.

### Background on COVID-19 pandemic and creation of shelters

The first human infection by severe acute respiratory syndrome coronavirus 2 (SARS-CoV-2), causing COVID-19, occurred in Wuhan, China.^10,11,12,13,14^ The virus spreads rapidly from human to human causing mild upper respiratory symptoms in some and severe pneumonia and organ failure in others with death occurring in 4.5% – 6.0% of the cases.^11,12,14^ The rapid spread, with no clinically approved antiviral drug or vaccine available to treat the condition, forced the World Health Organization (WHO) emergency committee to declare it a global pandemic.^11,12,14,15,16^

The South African government recognised the potential threat of a SARS-CoV-2 outbreak in February 2020, and the first case in South Africa was confirmed on 05 March 2020.^17^ On 15 March, a National State of Disaster for COVID-19 was declared.^18^ Initial national responses included a set of restrictions around travel and group gatherings and recommendations around hygiene and physical/social distancing. By the end of March, there were over 100 cases, with data pointing to an exponential increase of cases. On 27 March 2020, national lockdown in South Africa was announced in order to prevent the spread of COVID-19 that could affect millions of South Africans because of the high number of HIV, TB, poverty and malnourishment.^9,10,11^ A particularly vulnerable group was the large number of homeless individuals.

The initial national COVID-19 lockdown made it illegal to roam the streets and created the necessity for temporary homeless shelters in South Africa. Fifteen shelters were opened for the homeless in Tshwane on 29 March 2020. There was a rapid influx of homeless at these shelters, many needing medical attention. As a result of the lockdown, access to clinics and hospitals were severely restricted.^9,10,11^ In response to a request from COT, the DFMUP deployed family medicine registrars to assist with medical support at homeless shelters during COVID-19.

### Background to Community Oriented Substance Use Programme

The request from COT was made in the light of the existing capacity, experience and partnerships of the DFMUP Researching the development, application and implementation of a Community Oriented Substance Use Programme (COSUP) initiative that was in place since 2016. The COSUP is the first publicly funded, community-based programmatic response to the use of illegal substances in South Africa. It was founded on a system thinking, public health and clinical care harm reduction approach, and was implemented in partnership with the COT. The COSUP operates 17 service sites in four of the city’s seven regions as well as the DFMUP Community Orientated Primary Health Care (COPC) intervention areas. Service sites provide counselling, linkage to care and opioid substitution therapy services to 1513 adults who either smoke (51%) or inject (49%) heroin. The COPC healthcare workers link clinics to the surrounding community, doing home visits, counselling and education surrounding community on preventative health conditions. They help with child and woman health and help identify health needs in the community. Community Oriented Substance Use Programme, a programme that forms part of COPC, also offers needle and syringe services to people who inject drugs. The COSUP has established working relations with 169 organisations and has built human resource capacity in harm reduction among staff, clients and personnel within COSUP, DFMUP and in partner organisations.^3,10,11,19^

Community Oriented Substance Use Programme provides primary healthcare (PHC) and substance use services. The COT requested COSUP to support the delivery of medical support and integrated substance use services within the broader health and social system response to COVID-19. The national response to COVID-19 posed significant potential harm for people who use drugs, particularly efforts to reduce movement and placing people in (temporary) shelters as part of lockdown measures. Without access to opioid substitution therapy (methadone being mostly used locally, but suboxone also being an option at time), people who are dependent on heroin (an opioid that is known locally as nyaope, which is a mixture of heroin and cannabis^2,3,10,19,20,21^ will experience an involuntary period of abstinence, while at these shelters, which would result in withdrawal symptoms. Post release, without continued access to opioid substitution therapy, causes these people to be at a significant risk of overdose because of their reduced opioid tolerance.^3,10,20,21^ It was assumed that a large proportion of the residence would be substance users with increased risk of sexual transmitted diseases (STDs) like HIV, hepatitis and other.^2^ It was also assumed that there would be a large number of acute and chronic conditions among the homeless.^2^

### Aim

A descriptive, cross-sectional survey was used to describe the population of the shelters in the Tshwane District during the first COVID-19 lockdown as well as their current health, known and newly diagnosed diseases, and substance use patterns.

### Setting

This study reports on information gathered from the homeless who were allocated to the 15 different shelters that were created in the Tshwane District area. Most of the shelter sites were created at schools, churches and public stadiums with tents dormitories of various sizes set up for homeless people. Social development teams were in charge of the sites, and food was delivered by service providers contracted to the COT. Healthcare support to the sites was rendered by DFMUP as the nearby clinics did not have the capacity to accommodate the shelter population. Buses, tents or gazebos at the sites were used as clinics and medications were ordered from the nearest COT and Gauteng Department of Health (GDOH) clinics. The homeless people were all routinely screened for COVID-19, hypertension (HPT), diabetes mellitus (DM), HIV and TB in order to establish their health needs. They were also treated for the above conditions and any other acute illnesses that were reported or identified.

The homeless community was initially very unsure and scared; mostly worried they were going to get COVID-19 because of being forced to be at a shelter rather than living on the street with adequate social distancing. Some of the homeless community members with a history of substance use were also irritable as a result of withdrawal. As a result, some of them were hostile with the urge to abscond, but with time their trust was gained.

## Research method and design

All information was captured and analysed in Microsoft Excel 2010. The data analysis consisted of descriptive statistics such frequencies and proportions to describe the results. Specific focus was on grouping data according to age groups and investigating the information on substance use and chronic conditions. Data were divided into substance users and non-substance users as we wanted to know whether there is a difference in the disease profile among the groups.

The patients were screened for COVID 19 and TB on arrival and then allocated to a shelter bed accordingly. They were then made aware of the medical area at the shelters, where they could report any chronic or acute conditions in order to receive medical care. They were also asked if the information gathered during a medical consultation could be used for a study. The information was discarded and not used if the participant refused to the use of the information. They would get the same treatment whether they agreed to do so or not. They were not allowed to refuse TB or COVID 19 screening as this was for the safety of the population but could refuse any other screening. If they did screen, they were allowed to refuse that the information be used and were allowed to come for any medical treatment without it being used for research.

A cross sectional survey was done among the homeless people in the shelters to provide information of self-reported chronic conditions that the homeless populations at the shelters had during the lock down period. The participants were offered voluntary screening for medical conditions like HIV, HPT, DM, depression, etc. during the period of stay at the shelters. If HIV, HPT, DM, etc. was detected by screening tests, a formal blood work-up would be done via National Health Laboratory Services (NHLS).

### Study population and inclusion criteria

The study population included all residents of the homeless shelters in Tshwane. The total population in the 15 shelters was 2066 of which 1391 homeless people took part in the study. The information of all residents consenting to share their information were captured on a Microsoft Excel 2010 spread sheet. There were children at the shelters, but they were excluded.

### Data collection

Data were routinely collected and analysed by the medical staff and data capture teams on site while still attending to the needs of the shelter residents. Routine management of all shelter residents included: (1) screening for COVID-19, (2) taking of a medical history, (3) physical examination where necessary, and (4) establishing the medical needs of each individual. Initial health screening took place daily from 01 April to 30 July 2020.

Data were grouped according to different age groups. The information on substance uses and chronic conditions was captured on a Microsoft Excel 2010 spreadsheet.

### Ethical considerations

Ethical clearance to conduct this study was obtained from the Faculty of Health Sciences Research Ethics Committee of the University of Pretoria, South Africa (No: 310/2020). The study fell under a bigger study title: Researching the development, application and implementation of Community Oriented Substance Use Programme (COSUP) services during the response to the COVID-19 pandemic (83/2017).

To maintain confidentiality, the patient’s names were removed and replaced by a study number. The patients ID numbers were also replaced by yes for having an ID and no for not having an ID. The participants were then grouped into substance and non-substance users. The electronic files were stored on computers that were password protected.

This research did not place additional risk to clients or healthcare service providers as it focused on data collected to support service delivery. Clients voluntarily accessed health and social services, without renumeration. Instrument specific informed consent for research purposes was taken from clients and healthcare providers (clinicians, peers, allied professionals, service site managers, partner service providers). Any adverse events that took place in relation to health or psychosocial conditions were managed by the DFMUP team members that were allocated to different sites as per standard clinical practice. Staff were trained around maintaining client confidentiality and the certain risks people engaged in illegal practices may face as well as the principles of beneficence and non-maleficence.

## Results

The homeless shelter population had a total of 2066 individuals of which 1391 agreed to participate. The participants were 1305 (93.82%) African, 63 (4.53%) Caucasian, 20 (1.44%) coloured and 3 (0.22%) Indian. Of the 1391 homeless, 958 (68.87%) were South African, 92 (6.61%) were confirmed non-South African, and unfortunately 341 (24.51%) did not answer the question. The population consisted of 1333 (95.83%) men and 58 (4.17%) women. The results showed that most of the non-South Africans were from Zimbabwe, followed by Malawi, Mozambique, Kongo, Tanzania, Zambia, Uganda, Namibia, and Nigeria.

The population were asked if they have IDs, passports or some form of documentation. A total of 379 (27.25%) knew their ID number, eight (0.58%) claimed they did not have an ID, and 1004 (72.17%) did not answer the question.

The level of education was considered a reason for being homeless. It was found that 34.58% of the homeless participants had an education level above grade 10. Interestingly, it was found that 70.50% of the substance users that answered the question had completed grade 11 and 12. Unfortunately 54.57% of the participants did not want to answer the question on education level (Table 1).

**TABLE 1:** Education versus substance use versus chronic conditions.

Highest education	Males			Females		
	Number of males	Taking substances	Chronic conditions	Number of females	Taking substances	Chronic conditions
Grade 0–7	102	42	25	4	4	2
Grade 8–10	102	49	31	1	1	0
Grade 11–12	427	212	91	26	15	15
Tertiary education	1	-	-	-	-	-
Unknown	701	405	62	27	7	1

**Total**	**1333**	**708**	**209**	**58**	**26**	**18**

The mean age of the homeless participants was 35 years. It was found that 734 (52.77%) of the homeless used one or more substances with a mean age of 33 years, compared with 657 (47.23%) with a mean age of 37 years who did not use substances at all. The substance mostly used was heroin (80.38%) and cannabis (11.17%). Nyaope that is a combination of heroin mixed with cannabis and other substances like antiretroviral drugs were used by 34.8%.^2,10,19^ Other drugs used by the homeless were cocaine (5.18%) and crystal methamphetamines (4.09%). Unfortunately, alcohol use was only documented at one site and thus could not be used in the study.

The study showed that there was no difference in the prevalence of chronic conditions between the substances and non-substance users. Chronic conditions were prevalent in 16.24% (226 of 1391) of the homeless population. The most prevalent chronic conditions among the homeless were HIV at 9.56% (133), HPT at 2.52% (35), TB at 1.8% (25), epilepsy 1.22% (17), asthma 1.01% (14) and DM 0.65% (9). The prevalence of mental health condition was 2.23% (31). The mental health conditions found were schizophrenia 25.81% (8/31), insomnia 25.81% (8/31), bipolar mood disorder (BMD) 19.35% (6/31), depression 16.13% (5/31) and substance-induced psychotic disorder 6.45% (2/31). Of the 1391 participants, only 3.73% (50/1391) did not want their chronic conditions to form a part of the study. Table 2 gives a breakdown of the different age groups with the different chronic conditions. Substance users and non-substances users were compared, and the totals for combined group can be found at the bottom of the Table 2.

**TABLE 2:** Prevalence of disease among the homeless.

Age groups (years)	Total in group	Unknown	No chronic condition	Chronic conditions	HIV	TB	Hepatitis	HPT	DM	Asthma	Epilepsy	OA	Mental health
**Substance use**													
< 20	0	0	0	0	0	0	0	0	0	0	0	0	0
20–30	293	28	231	34	25	5	3	2	0	2	1	1	1
31–40	331	15	264	49	39	5	4	3	1	2	4	0	4
41–50	68	3	51	14	11	0	0	2	0	0	0	0	0
51–60	24	0	12	12	5	1	0	5	0	0	1	0	2
> 60	7	0	7	0	0	0	0	0	0	0	0	0	0
Unknown age	11	4	6	1	1	0	0	0	0	0	0	0	0
Sub-total	734	50	571	113	81	11	7	12	1	4	6	1	7
**No substance use**													
< 20	3	0	3	0	0	0	0	0	0	0	0	0	0
20–30	156	2	136	18	9	2	0	1	1	3	2	0	6
31–40	264	10	222	32	22	5	0	3	3	1	3	3	5
41–50	119	3	85	31	18	5	0	3	0	3	3	4	3
51–60	54	5	33	16	2	2	0	10	3	2	2	0	3
> 60	27	1	15	11	1	0	0	6	1	1	0	2	4
Unknown age	34	1	28	5	0	0	0	0	0	0	1	1	3
Sub-total	657	22	522	113	52	14	0	23	8	10	11	10	24

**Total**	**1391**	**72**	**1093**	**226**	**133**	**25**	**7**	**35**	**9**	**14**	**17**	**11**	**31**

HIV, human immunodeficiency virus; TB, tuberculosis; HPT, hypertension; DM, diabetes mellitus; OA, osteo-arthritis.

## Discussion

The homeless population is a vulnerable community susceptible to physical and mental health problems.^1,2,3,8^ They are a poorly understood population with only a few research studies conducted in an attempt to understand the population.^3,5,7^ Studies on the population are needed because of their socio-political and economical vulnerability.^8^ The population is also thought of as being homeless because of loss of contact with families^5,7^ and their resistance to assistance.^7^

The lockdown period was an ideal opportunity to gather information on the population, and it was expected to result in a large population of over 10 000. According to the 2011 Statistics South Africa (SSA) figures, there were 6244 street-homeless people in the COT, of which 3747 people were concentrated in region 3.^2,8,10,11^ Unfortunately only a total of 2066 homeless were placed in the shelters and only 1391 of the study population agreed to participate. This finding could possibility indicate that street-homeless are less than originally thought, as the rest must have stayed somewhere else during lockdown. The other possibility is that some of the homeless might have migrated back to their point of origin or that they were re-united with families by the social services and law enforcement efforts.

In the study conducted, it was found that only 27.25% of the homeless participants had identifying documentation and 72.17% did not want to answer the question, which either means they did not have the documentation or that they do not trust any form of administrative processes.

There was an attempt by Cross et al.^7^ to study the demographics of the homeless that had similar findings determining that the homeless did not want to co-operate with providing information, did not have good relationship with administrative offices, and did not have appropriate documentation in the form of identification documents or passports.^1,7^

Demographics results of the study done showed that being homeless affects South African and non-South Africans of all races, with the majority being African. Unfortunately, a quarter of the study population (24.51%) did not answer the question, which prevents 100% accuracy of the result found. Fortunately, Kok et al.^5^ and Cross et al.^7^ who had conducted separate studies on the homeless also discovered that being homeless affects people of all races in South Africa, but with the largest part of the population being South Africans and non-South Africans,^1,5,7,8^ which is in line with the findings in the study.

In the study, it was found that 32.57% had an education higher than Grade 10 and only 15.03% had an education level below Grade 10. Unfortunately, 52.34% did not want to provide the information. According to Education at a Glance 2019,^22^ 59% of South Africans between age 25 and 34 had passed Grade 12 in 2018. The homeless participants level of education was considered a possible variable for substance use, with the idea that with lower education comes higher risk of substance use. Out of the 322 substance users that answered the question, it was found that 227 (70.50%) of them completed grades 11 and 12 (Table 1). This may indicate that leaving school early does not put you at risk for substance use, but this could be a false finding because of the fact that more than 50% of the participants, using substances, did not want to answer the question. They might not have wanted to answer the question because of feeling intimidated by having low grades. Kok et al.^5^ had a different finding that showed that the homeless population generally do have an education but are unable to enter the job market with an incomplete Grade 10 and/or higher.^5^

Information on the health needs of the homeless is limited worldwide.^1,2^ It has been reported that the reason might be that the homeless find it difficult to access healthcare because of travel challenges faced reaching the facilities and also being treated poorly at health institutions from their onset of arrival.^1,2^ They also do not have proof of residential address or identification documentation that worsens the situation.^2^ This was confirmed by one of the shelters, next to a clinic, that refused to see the patients because of work load, staff insecurity, poor documentation of homeless, etc. This forced a medical team from University of Pretoria to step in and attend to the health needs of the homeless at this site.

An journal article on the profile of the homeless in Johannesburg in 2016 determined that 74.16% of the population used some form of drug.^1,2^ A homeless survey conducted in 2010 and 2019 showed similar results indicating that Cannabis was one of the main substances used with a 5.4% – 18.5% prevalence followed by Mandrax (methaqualone combined with diphenhydramine), heroin, tik (crystal methamphetamine) and ecstasy (MDMA) that had a 5% prevalence among the homeless, a lot less than expected.^1,2^ In our study, 52.77% of our total population studied used substances that was less than the 2016 result. It was found that heroin was the main substance of use followed by cannabis and then cocaine and crystal methamphetamines. The lower reported substance use percentage might be because of the fact that the population did not need medical intervention for it. The higher heroin substance use findings could be related to the fact that the population needed medication to prevent withdrawal and were therefore more inclined to disclose the information.

The use of substance with the lack of access to hygiene facilities and exposure to the elements are usually linked to risky behaviour. Risky behaviours like sharing of needles plus higher incidence of promiscuity under the influence of substances may lead to the possibility of higher incidences of TB, HIV, hepatitis and other STDs.^1^ The prevalence of known status and newly diagnosed TB and HIV among the homeless participants studied were 1.29% and 6.89%, respectively. The prevalence of TB found in a 2019 homeless study was similar, showing 1.2% – 2.5% of homeless that had TB, but the HIV result was much less compared with 17.4% found in a 2019 homeless study.^2^ The low percentage of TB might suggest that they were not really at risk of contracting COVID-19 if there was no shelters during lockdown period as they were already practicing social distancing, but rather would have become ill as a result of lockdown, because of not having access to food during this period. The fact that they were placed in a condensed shelter actually increased their risk for contracting COVID-19. Luckily, there was a respiratory area at the shelters where TB patients were kept separate from the other population, and there was also a COVID-19 tent that was luckily not needed at the shelters.

The current study showed that there was no difference in the prevalence of known and newly diagnosed chronic conditions between the substances and non-substance users. The study also showed much less chronic conditions as expected when comparing results with a homeless health survey of 2019 and the district health barometer 2018/2019. The homeless health surveys of 2019 found that 16% of the population had HPT.^1,2^ The 2018/2019 district health barometer showed a 20.7% prevalence of HPT and 10.6% prevalence of DM.^23^ Mental health findings in the study of 1.6% was similar to prevalence of mental health findings of 2.5% in the district health barometers of 2018/2019,^23^ but way less than the homeless health survey of 2019 that showed that 58% of the population had some form of mental health conditions.^1,2,23^ There are a few reasons that the findings in the study could have been less. Possible reasons could be that not everyone wanted to be screened for these conditions; some might have felt they feel fine and don’t need their medication anymore; they might have just shared information that was needed to survive at the shelters, for example, heroin users who needed methadone.

### Study limitations

Information could not be gathered from all the sites, as some participants were not willing to provide the required information. However, this fell within their ethical right, and there was no compensation for this limitation. Information was also self-reported, which leads to the possibility of major response bias as there was, for example, no proof that a participant achieved his level of education and patients could have not been honest on other substance use they had. Another limitation to the study is that the information was analysed after the shelters had closed so we could not go back to find out if the population that did not want to join felt more comfortable to join the study and also could not go back to ask whether the population that took part feels more comfortable to give the missing information. It was also found that only one shelter kept record of known and newly diagnosed chronic conditions. There was also no record kept of previously defaulted chronic conditions. It was also found that some shelters added alcohol as a substance and others did not so we could not use the data, which is a prominent substance to look at and should have been considered. The population were given a linkage to care booklet to be able to continue care at clinics after lockdown but were unable to follow up if they continued treatment or not.

### Recommendations

World Health Organization’s definition of health is a state of complete physical, mental and social wellbeing and not merely the absence of disease or infirmity.^1,3^ It is recommended that more be done to address the needs of the homeless population and efforts should be directed to help improve the social economic status of the homeless in order to improve the general health of the community. One of the needs is to improve awareness of the health needs of the homeless and incorporate it in the training of health professionals, so as to encourage more sympathetic approaches to the population.^1^

There are multiple rehab sites for substance use, but emphasis should be made on whether all the sites are in order for the community to be able to use the services to its maximum capacity. Clinics and hospitals should try and find a way to accommodate a patient’s medical needs even though he or she does not have a fixed address. The Link to Care booklet used by some hospitals in the Tshwane area is a great idea to help improve access to clinics for a mobile homeless population we have in the country. The booklet could be used and considered by the rest of the country so that should the patient find himself or herself at a new clinic they would be able to show the clinic their treatment, and the clinic or hospital will also be able to follow up the NHLS results with the file number of the previous institution.

It would also be recommended for clinics and/or hospitals to reach out to homeless shelters in their areas in order to improve health services to this population.

Building trust outside of an enforced shelter setup would improve strategies to address their complete health state within the health services in this community.^2,3^ This includes addressing stereotypical mind sets and treatment by health staff, improving chronic condition identification and control, improving access to dental services and by promoting health education towards this vulnerable population.^2,3^ Non-governmental organisations (NGOs) and public healthcare systems should also be put in place to provide sustainable services that are accessible to this community.^2,3,10,11^ A recommendation to use mobile clinics to visit homeless people where they are might be useful. This will improve knowledge on their health challenges, access to health services and also openness with their personal information. These types of relationships will not only improve health of the population but also provide research opportunities to better understand the community.

## Conclusion

As found in previous studies, homelessness is not a simple matter of poverty.^7^ The findings indicate that there is still a lot to be learned from the homeless populations’ health, which will only be feasible if the Department of Health, Social Welfare, Housing and Labour and the many other non-governmental and faith-based organisations work together to serve the population rather than turning a blind eye.^1,3^

One of the factors to be addressed is building trust with this specific population as in the study there was multiple times that information was not shared. There was specifically a large part of the group that did not want to divulge personal information like citizenship and education, which was in line with previous studies findings. Information bias might have been made worse by the fact that the population was forced to be in shelters during this lockdown period.

Half the homeless population were found to use some form of substance. They are generally educated and had similar TB and HIV prevalence compared with the rest of the population but had lower rates of HPT and DM. The study missed the opportunity to test compliance to treatment and to follow up default rates of the population. It also failed to asked if the population has difficulties reaching clinics or hospitals and if they felt it necessary to be screened and treated for chronic conditions.

Efforts should be made to improve education, and research around the homeless population, by government and non-government facilities by building relationships with homeless shelters in their areas outside of a lockdown setup is needed to focus on the population to help improve their physical, spiritual, emotional and socio-economic health.
